# Supplementary material for: Reactive P and S co-doped porous hollow nanotube arrays for high performance chloride ion storage
Source: Nat Commun. 2024 Jun 10;15:4951. doi: 10.1038/s41467-024-49319-5 (PMC11164705; doi:10.1038/s41467-024-49319-5)
Supplement: Supplementary file 1 — Supplementary Information [file 41467_2024_49319_MOESM1_ESM.pdf]

Supplementary Information

Reactive P and S co-doped porous hollow nanotube arrays for high performance chloride ion storage

Siyang Xing[1,2], Ningning Liu[1], Qiang Li[1], Mingxing Liang[1,3,4], Xinru Liu[1,5], Haijiao Xie[6], Fei Yu[7], Jie Ma[1,8*]

1 Research Center for Environmental Functional Materials, State Key Laboratory of Pollution Control and Resource Reuse, College of Environmental Science and Engineering, Tongji University, Shanghai, 200092, P. R. China.

2 Department of Energy, Environmental & Chemical Engineering, Washington University in St. Louis, St. Louis, Missouri 63130, United States

3 College of Chemistry and Environmental Engineering, Shenzhen University, Shenzhen 518060, P.R. China

4 College of Physics and Optoelectronic Engineering, Shenzhen University, Shenzhen 518060, P.R. China

5 School of Architecture, Civil and Environmental Engineering, EPFL, Lausanne, Vaud1015, Switzerland

6 Hangzhou Yanqu Information Technology Co., Ltd., Y2, 2nd Floor, Building 2, Xixi Legu Creative Pioneering Park, No. 712 Wen'er West Road, Xihu District, Hangzhou 310003, P. R. China

7 College of Oceanography and Ecological Science, Shanghai Ocean University, No 999, Huchenghuan Road, Shanghai, 201306, P. R. China

8 School of Civil Engineering, Kashi University, Kashi 844000, China

The file includes Supplementary Figs. 1-14, and Supplementary Tables 1-4.

Supplemental Figures

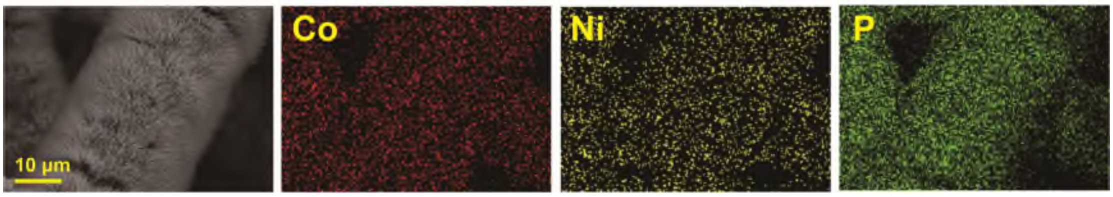

Supplementary Fig. 1｜ SEM images of CoNiP@CF and corresponding elemental distribution maps.

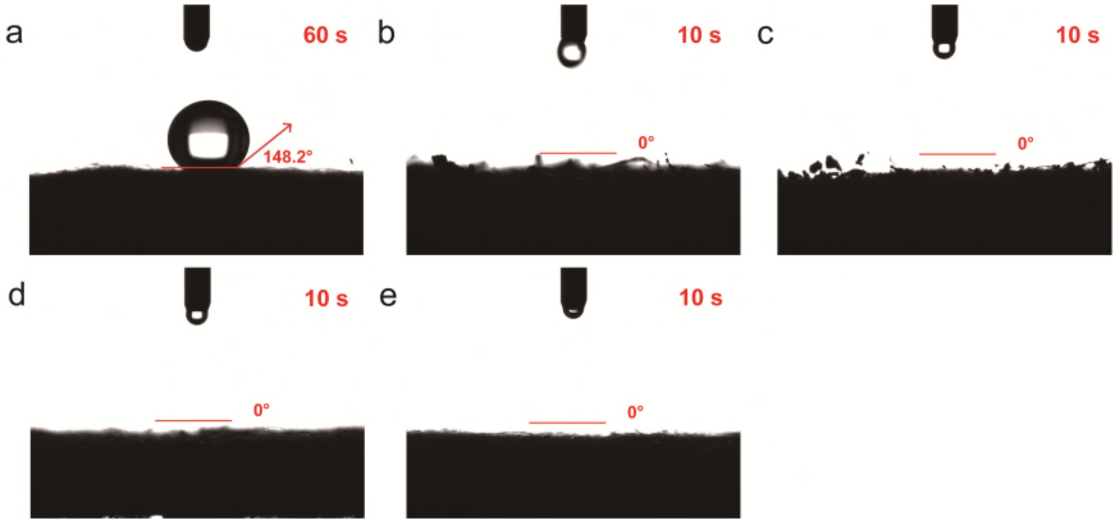

Supplementary Fig. 2│ The optical image of water contact angle on the surfaces of (**a**) CF, (**b**) pCF, (**c**) CoNiOH@CF, (**d**) CoNiP@CF and (**e**) CoNiPS@CF.

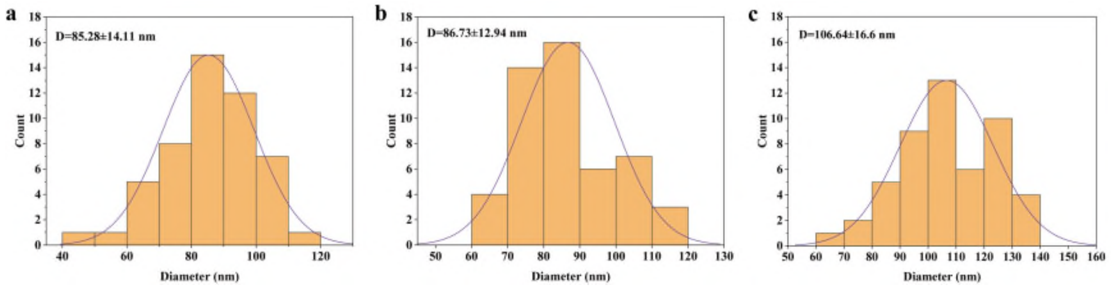

Supplementary Fig. 3 | Diameter distribution of (**a**) CoNiOH@CF, (**b**) CoNiP@CF nanoneedles and (**c**) CoNiPS@CF nanotubes from SEM.

From the SEM images, we can see the changes in nanoneedle diameter before and after different doping processes. We counted the diameters of the 50 nanoneedles in the SEM pictures. The statistical results are basically consistent with the normal distribution. From the results, we can see that the diameter of CoNiOH is about 85.28 ± 14.11 nm, which is slightly smaller than the diameter of CoNiP (86.73 ± 12.94 nm). This is because during the chemical vapor deposition (CVD) process, the initial transition metal hydroxide is doped with phosphorus to form the transition metal phosphide, so its diameter also changes. In addition, the advantage of the CVD method is that it has little change in the original electrode morphology and can maintain its original high specific surface area nanoneedle morphology. However, when we further doped sulfur, the surface nanoneedle morphology changed, and porous nanotubes were gradually formed from the nanoneedles. Therefore, the average diameter of CoNiPS is the largest among the three samples, which is 106.64 ± 16.6 nm.

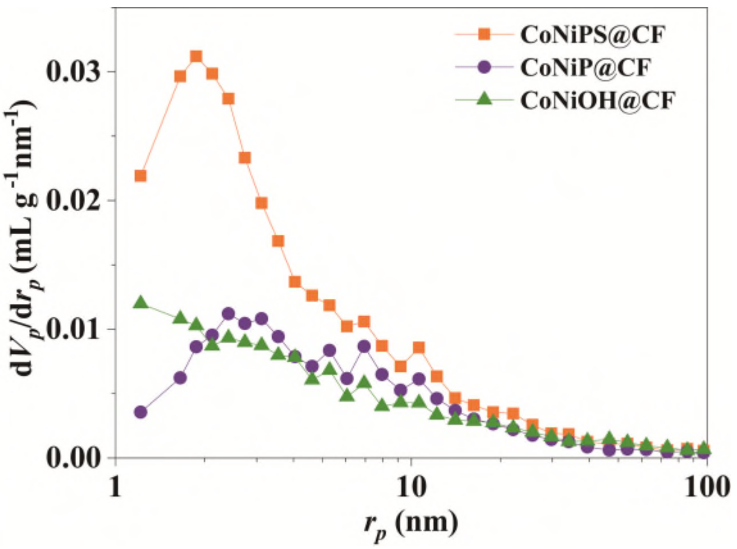

Supplementary Fig. 4 | BJH pore size analysis of different electrodes.

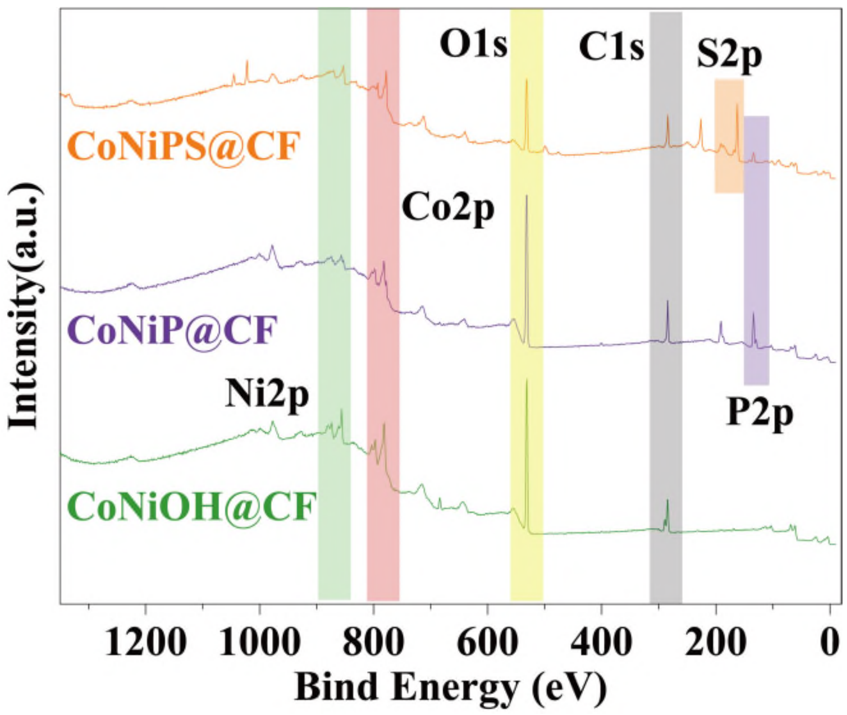

Supplementary Fig. 5| XPS full spectra of different electrodes.

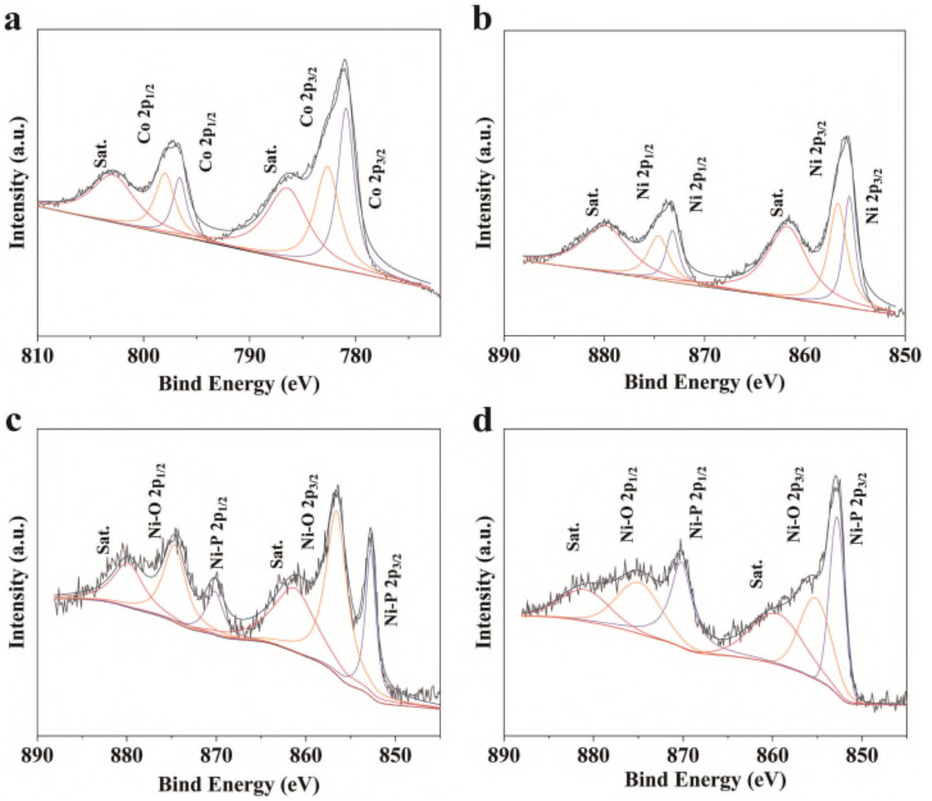

Supplementary Fig. 6 | **a** High-resolution Co 2p spectra of CNOH@CF and (**b, c** and **d**) Ni 2p spectra of CNOH@CF, CNP@CF and CNPS@CF.

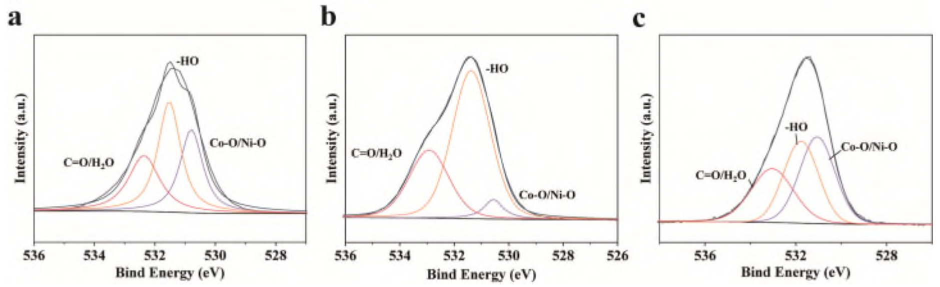

Supplementary Fig. 7 | High-resolution O 1*s* spectra of (**a**) CNOH@CF, (**b**) CoNiP@CF and (**c**) CoNiPS@CF.

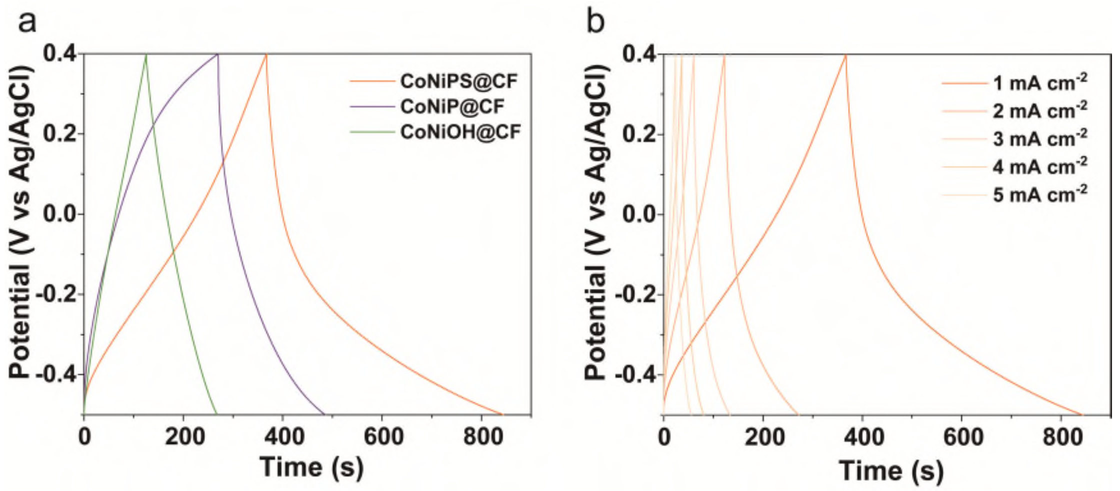

Supplementary Fig. 8 | **a** The galvanostatic charge-discharge (GCD) profiles of different samples at 1 mA cm^{-2}; b GCD profile of CoNiPS@CF electrode at different current density.

Galvanostatic Charge-Discharge (GCD) experiments were conducted on all samples at a current density of 1 mA cm^{-2}. Among the samples, CoNiPS@CF demonstrated the highest specific capacitance of 0.103 mAh cm^{-2}, which is significantly higher than CoNiOH@CF and CoNiP@CF, with specific capacitance of 0.075 mAh cm^{-2}, and 0.034 mAh cm^{-2}, respectively. Furthermore, no plateau was observed at different specific currents, suggesting that the CoNiPS@CF samples work through a pseudocapacitive mechanism, like other transition metal oxides and hydroxides. Similar conclusions can also be drawn from cyclic voltammetry test, representing a quasirectangular shape without observable redox peaks.

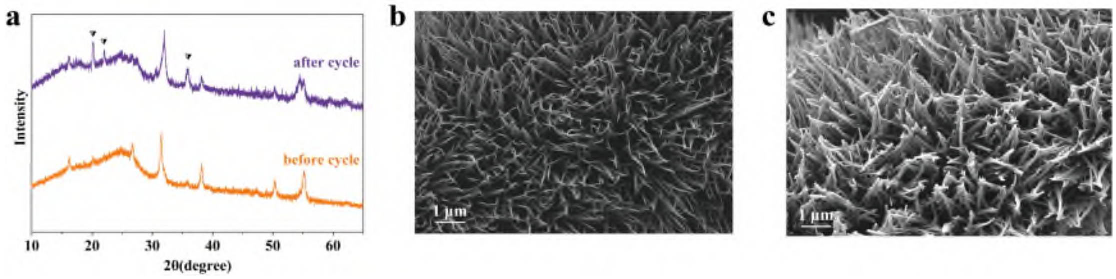

Supplementary Fig. 9 a XRD of samples before and after 500 CV cycles. Electrode morphology of CoNiPS@CF **(b)** before and **(c)** after 500 CV cycles.

From the results of XRD, it can be seen that the XRD of the sample after 500 CV cycling is still basically consistent with the original sample. But there are still some new crystal phases formed: at $2\theta = 20.16$ °, this is the crystal phase of CoO(OH), which represents the oxidation phenomenon caused by the partial reaction between the surface electrode and the electrolyte during the adsorption process; at $2\theta = 21.98$ ° and 35.97 °, it shows that the adsorption and desorption of ions on the electrode surface leads to the destruction of the original crystal phase and the formation of new crystal phases ($P_4O_6S_2$ and $Ni_2P_2S_6$). However, even with the presence of these new crystal phases and a certain degree of oxidation, there was no obvious decline for desalination performance. So the high retention of electrochemical capacities for the electrode contributed to the excellent cycling performance due to the partial corrosion to a low extent.

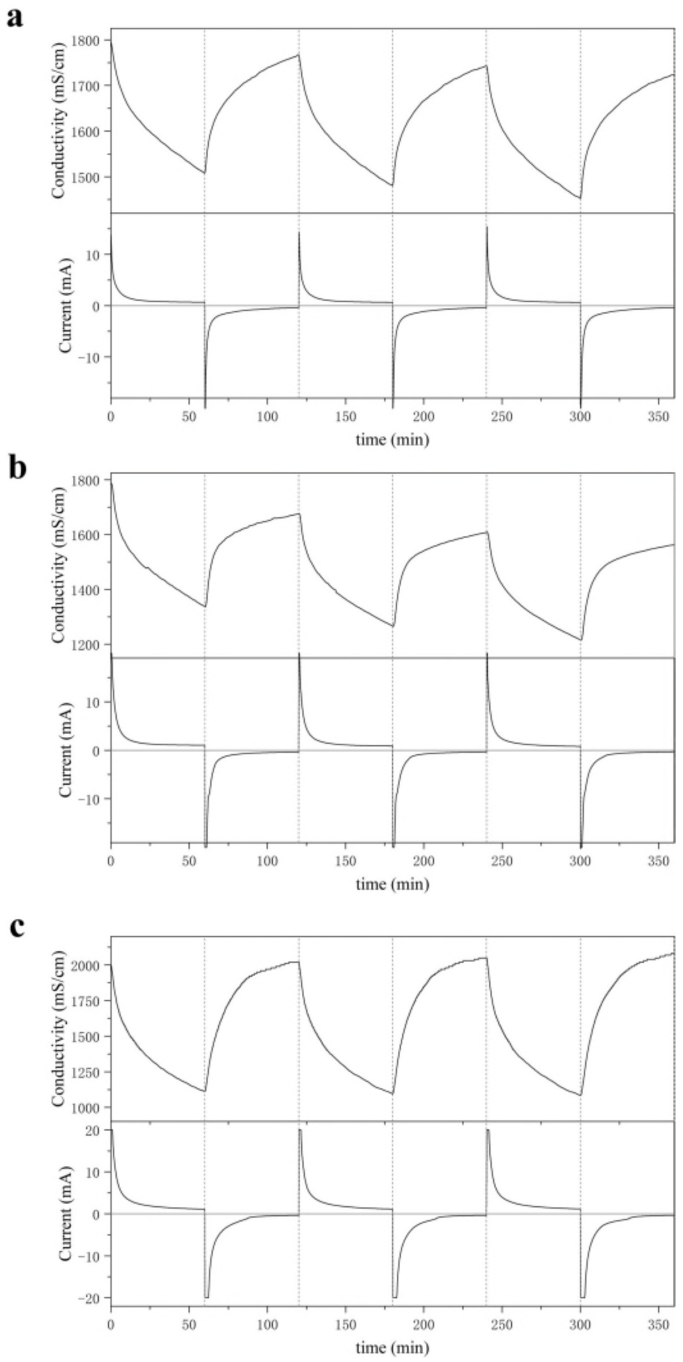

Supplementary Fig. 10 | Conductivity and current versus time images of (**a**) CoNiOH@CF, (**b**) CoNiP@CF and (**c**) CoNiPS@CF

We have incorporated data illustrating the evolution of conductivity and current over time in a NaCl solution with an initial concentration of ~1000 mg/L under a constant voltage of 1.2V for three cycles. Compared to the other two samples, the desorption capacity of CoNiP is notably smaller than its adsorption capacity. This is due to the higher adsorption energy of Cl^- by CoNiP, resulting in irreversible capacity loss and a high ion diffusion barrier. Consequently, active adsorption sites may gradually

deactivate during the experiment, diminishing the electrode's adsorption capacity. This results in the inability of the electrode to sustain optimal performance over an extended period, thereby reducing cycle stability. Furthermore, ion adsorption will induce a certain degree of bulk phase expansion. The fact that the desorption capacity is lower than the adsorption capacity indicates that certain ions on the surface are adsorbed into the electrode layers and cannot be effectively desorbed. This exacerbates bulk phase expansion, which in turn impacts the structural stability of the electrode. In contrast, CoNiPS@CF electrode showed a decent reversibility, and the desorption capacity and adsorption capacity are basically equal, which can be attributed to the lower Cl^- adsorption energy of CoNiPS@CF. And it has the highest specific adsorption capacity, with an average SAC of 71.4 ± 4.3 mg_{Cl-} g^{-1}.

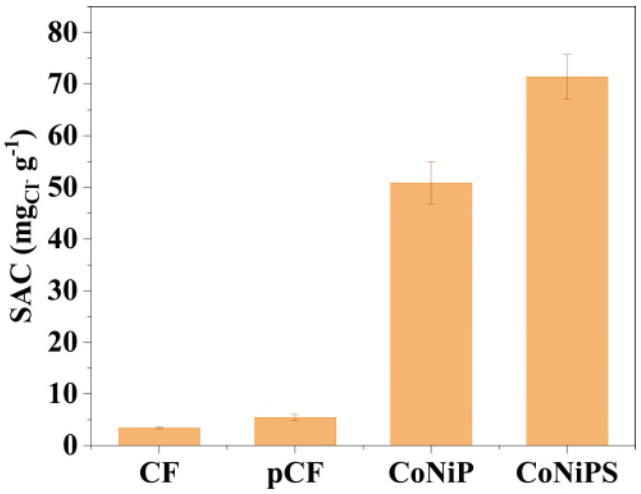

Supplementary Fig. 11 | SAC comparison between CF, pCF, CoNiP@CF and CoNiPS@CF at 1.2V (1000 mg L^{-1} NaCl solution). Error bars are means ± standard deviation (n=4 replicates). Error bars are means ± standard deviation (n=4 replicates).

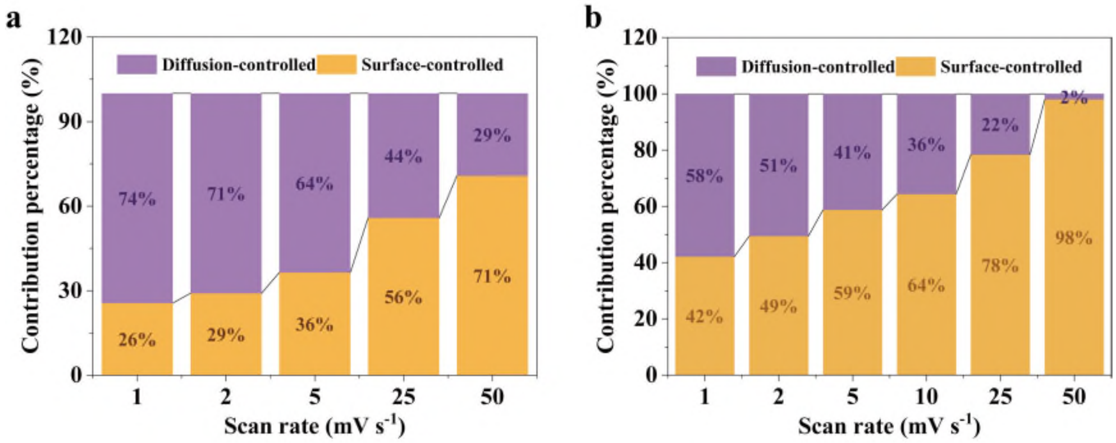

Supplementary Fig. 12│ Normalized contribution ratios of surface-/diffusion-controlled capacities of **(a)** CoNiP@CF and **(b)** CoNiPS@CF.

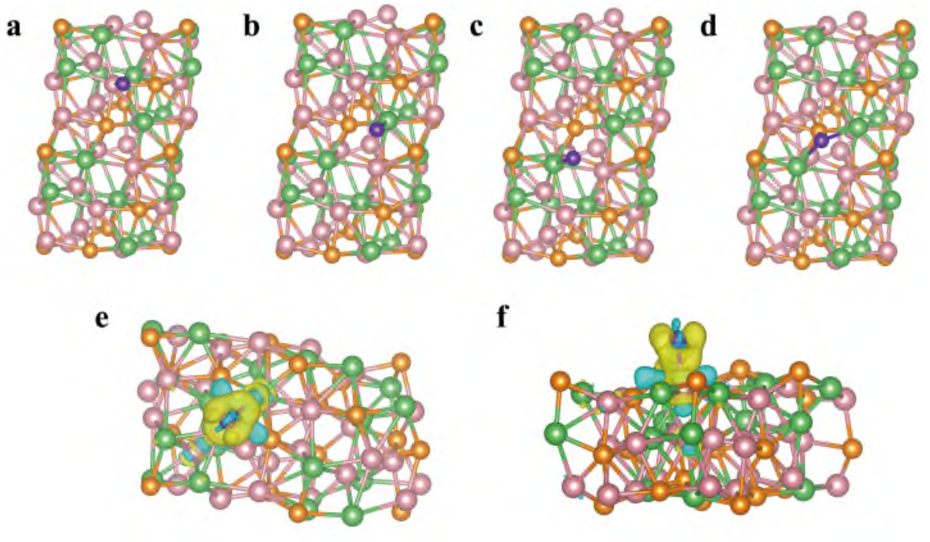

Supplementary Fig. 13 | Top view of relaxed adsorption configurations for corresponding E_{ads} for Cl^- of Co(**1 site, a**), Ni1(**2 site, b**), Ni2 (**3 site, c**) and P (**4site, d**) on CoNiP. **e f** The difference charge density for Ni1(**2 site, b**) to Cl^-. Yellow represents gain of electrons and blue represents loss of electrons.

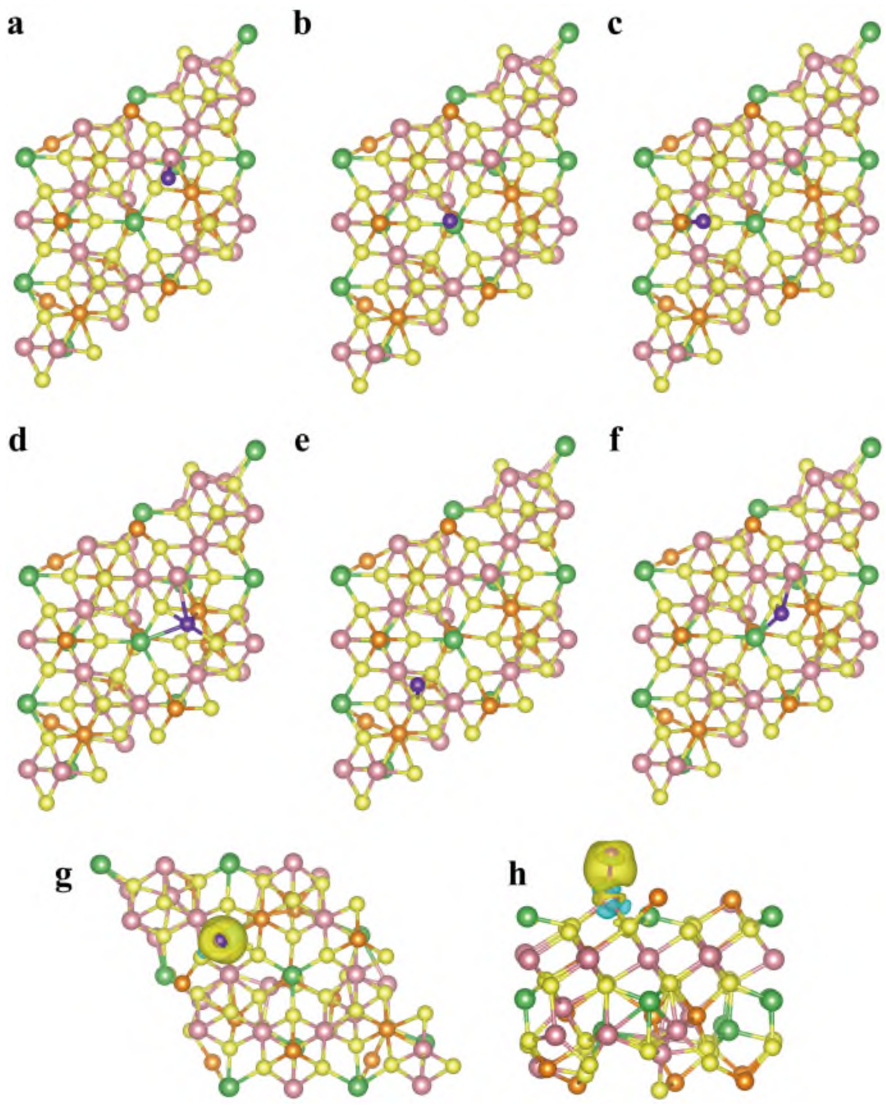

Supplementary Fig. 14 | Top view of relaxed adsorption configurations for corresponding E_{ads} for Cl^{-} of Co(**α site, a**), Ni(**β site, b**), P1 (**γ site, c**), P2 (**δ site, d**), S1 (**ε site, e**) and S2 (**η site, f**) on CoNiPS. **g, h** The difference charge density for Co (**α site, a**) to Cl^{-}. Yellow represents gain of electrons and blue represents loss of electrons.

We have employed the plane-wave code Vienna ab-initio simulation package (VASP) program to perform all the spin-polarized density functional theory (DFT) calculations within the generalized gradient approximation (GGA) using the Perdew-Burke-Ernzerhof (PBE) formulation. We have chosen the projected augmented wave (PAW) potentials to describe the ionic cores and take valence electrons into account using a plane wave basis set with a kinetic energy cutoff of 450 eV. Partial occupancies of the Kohn−Sham orbitals were allowed using the Gaussian smearing method and a width of

0.05 eV. The Brillouin zone was sampled with Monkhorst mesh of 2×2×1 for the optimization for all structures. The self-consistent calculations apply a convergence energy threshold of 10^{-5} eV, and the force convergency was set to 0.05 eV/Å.

The adsorption energy (E_{ads}) of an adsorbate A was defined as：

$E_{ads} = E_{Cl^-/slab} - E_{slab} - E_{Cl^-}$

where $E_{Cl^-/slab}$, E_{slab} and E_{Cl^-} are the energy of the Cl^- adsorbed on the surface slab, the energy of surface slab, and the energy of Cl^- respectively.

Supplementary Tables

Supplementary Table 1. Ion exchange membrane parameter information

Parameter	CEM8040	AEM8040
Switching Capacity (mol kg^{-1})	2.2	2.0
Selective Permeability (%) ≥	95 %	95 %
Acid-base Tolerance Concentration (mol L^{-1}) ≤	3 mol L^{-1}	3
Wet-state Thickness (mm)	0.30 – 0.35	0.16 – 0.23
Membrane Resistance (Ω cm^2 ,0.5M NaCl, 25℃) ≤	5.5	4.0
Current Density (A m^{-2}) ≤	400	300
Water Permeability (mL h^{-1} cm^{-2} MPa^{-1}) ≤	0.1	0.1
Thermal Stability (℃) ≤	50	50

Supplementary Table 2. Surface area, pore size, and pore volume of different electrodes.

Electrodes	BET surface area (m^2 g^{-1})	Pore volume (cm^3 g^{-1})	Pore size(nm)
CF	2.4336	0.00362	5.9555
CoNiOH@CF	39.525	0.17	17.206
CoNiP@CF	50.135	0.1846	14.728
CoNiPS@CF	92.975	0.2747	11.819

Supplementary Table 3. R_{int} and R_{ct} of electrodes before and after long cycling.

Electrodes	Before long cycle		After long cycle	
	R_{int}	R_{ct}	R_{int}	R_{ct}
CNP@CF	3.704	3.882	3.624	4.261
CNPS@CF	2.002	3.386	2.396	3.138

Supplementary Table 4. Comparison of SAC of various CDI electrodes.

Electrode	Applied Voltage/Specific Current	Salinity (mg L^{-1})	SAC (mg g^{-1})	SAR (mg g^{-1} min^{-1})	Energy Consumption (kWh kg^{-1})	References
CoNiPS@CF	1.2 V	1000	125.33	6.96	0.161	This work
CNF-4	1.2 V	800	63.51	4.38	1.07	[ref][1]
HCl-NC	1.2 V	1000	100.3	~1.75	0.394	[ref][2]
NC@GC/CNTs	1.4 V	1000	37.35	~3	-	[ref][3]
Electrospun PCF	1.0 V	500	30.4	~3	0.366	[ref][4]
PPy-p-TS//PPy-ClO_4	0.75 V	585	74.3	2.46	0.288	[ref][5]
PB/PANI	100 mA g^{-1}	500	133.3	1.8	~1.01	[ref][6]
NVOPF/rGO	50 mA g^{-1}	1000	~115	~2.24	0.35	[ref][7]
Ag/AgCl	0.2 V	292500	85	0.3	-	[ref][8]
Na-FeOOH//Cl-FeOOH	1.2 V	500	35.12	0.3	-	[ref][9]
MnO_{2-x}	1.2 V	500	62	~4	-	[ref][10]
NVP/GA	1.4 V	1000	107.5	2.1	-	[ref][11]
FeOOH/Pd/MoS_2	1.2 V	500	41.1	3.6	-	[ref][12]
Porous $Ti_3C_2T_x$	1.2 V	10000	118	-	-	[ref][13]
mPDA/MXene	1.5 V	1000	37.72	1.27	0.69	[ref][14]
Bi-ene NSs@MXene	1.2 V	1170	88.2	~9	0.78	[ref][15]
N-$Ti_3C_2T_x$	1.2 V	5000	43.5	-	-	[ref][16]
Functionaltion Mxene	1.2 V	5000	49	2.92	0.38	[ref][17]
$W_{18}O_{49}$/Ti_3C_2	1.2 V	500	29.25	0.97	0.564	[ref][18]
TiO_2/Ti_3C_2	15 mA g^{-1}	500	75.62	0.68	0.49	[ref][19]

Supplementary Reference

1. Liu R, Wang Y, Wu Y, Ye X, Cai W. Controllable synthesis of nickel–cobalt-doped Prussian blue analogs for capacitive desalination. *Electrochim Acta*, 141815 (2023).
2. Liang MX*, et al.* A reverse-defect-engineering strategy toward high edge-nitrogen-doped nanotube-like carbon for high-capacity and stable sodium ion capture. *Adv Funct Mater* **32**, 2209741 (2022).
3. Zhang Y*, et al.* MOF-on-MOF nanoarchitectures for selectively functionalized nitrogen-doped carbon-graphitic carbon/carbon nanotubes heterostructure with high capacitive deionization performance. *Nano Energy* **97**, 107146 (2022).
4. Liu T*, et al.* Exceptional capacitive deionization rate and capacity by block copolymer–based porous carbon fibers. *Sci Adv* **6**, eaaz0906 (2020).
5. Huang H-Y, Tu Y-H, Yang Y-H, Lu Y-T, Hu C-C. Dopant-designed conducting polymers for constructing a high-performance, electrochemical deionization system achieving low energy consumption and long cycle life. *Chem Eng J* **457**, 141373 (2023).
6. Shi W*, et al.* Enabling superior sodium capture for efficient water desalination by a tubular polyaniline decorated with prussian blue nanocrystals. *Adv Mater* **32**, 1907404 (2020).
7. Xing S, Cheng Y, Yu F, Ma J. $Na_3(VO)_2(PO_4)_2F$ nanocuboids/graphene hybrid materials as faradic electrode for extra-high desalination capacity. *J Colloid Interface Sci* **598**, 511-518 (2021).
8. Ahn J*, et al.* High performance electrochemical saline water desalination using silver and silver-chloride electrodes. *Desalination* **476**, 114216 (2020).
9. Zhao J*, et al.* Efficient and durable sodium, chloride-doped iron oxide-hydroxide nanohybrid-promoted capacitive deionization of saline water via synergetic pseudocapacitive process. *Adv Sci* **9**, e2201678 (2022).
10. Fu Z*, et al.* Local Electric Field Induced by Atomic-Level Donor-Acceptor Couple of O Vacancies and Mn Atoms Enables Efficient Hybrid Capacitive Deionization. *Small*, e2205666 (2023).
11. Zhao W, Ding M, Guo L, Yang HY. Dual-Ion Electrochemical Deionization System with Binder-Free Aerogel Electrodes. *Small* **15**, 1805505 (2019).
12. Zhao Z, Zhao J, Sun Y, Ye M, Wen X. Synergetic pseudocapacitive sodium capture for efficient saline water desalination by iron oxide Hydroxide-Decorated palladium nanoparticle anchored 3D flowerlike molybdenum sulfide. *Chem Eng J* **458**, 141508 (2023).
13. Bao W*, et al.* Porous Cryo-Dried MXene for Efficient Capacitive Deionization. *Joule* **2**, 778-787 (2018).
14. Li Q*, et al.* Two-dimensional MXene-polymer heterostructure with ordered in-plane mesochannels for high-performance capacitive deionization. *Angew Chem Int Ed Engl* **60**, 26528-26534 (2021).
15. Gong S*, et al.* Vertically Aligned Bismuthene Nanosheets on MXene for High-Performance Capacitive Deionization. *ACS Nano* **17**, 4843-4853 (2023).
16. Amiri A, Chen Y, Bee Teng C, Naraghi M. Porous nitrogen-doped MXene-based electrodes for capacitive deionization. *Energy Stor Mater* **25**, 731-739 (2020).
17. Bo Z*, et al.* Anion-kinetics-selective graphene anode and cation-energy-selective MXene cathode for high-performance capacitive deionization. *Energy Stor Mater* **50**, 395-406 (2022).
18. Liang J, Yu J, Xing W, Tang W, Tang N, Guo J. 3D interconnected network architectures

assembled from $W_{18}O_{49}$ and Ti_3C_2 MXene with excellent electrochemical properties and CDI performance. *Chem Eng J* **435**, 134922 (2022).

19. Liu N, *et al.* Ti_3C_2 -MXene partially derived hierarchical 1D/2D TiO_2 /Ti_3C_2 heterostructure electrode for high-performance capacitive deionization. *Adv Sci* **10**, e2204041 (2023).
